# mGluR1-Dependent Long Term Depression in Rodent Midbrain Dopamine Neurons Is Regulated by Neuregulin 1/ErbB Signaling

**DOI:** 10.3389/fnmol.2018.00346

**Published:** 2018-10-01

**Authors:** Ada Ledonne, Nicola Biagio Mercuri

**Affiliations:** ^1^Department of Experimental Neuroscience, IRCCS Santa Lucia Foundation, Rome, Italy; ^2^Department of Systems Medicine, University of Rome Tor Vergata, Rome, Italy

**Keywords:** neuregulin 1, ErbB signaling, group 1 metabotropic glutamate receptors (mGluRI), synaptic plasticity, LTD, dopamine, substantia nigra

## Abstract

Increasing evidence demonstrates that the neurotrophic factor Neuregulin 1 (NRG1) and its receptors, ErbB tyrosine kinases, modulate midbrain dopamine (DA) transmission. We have previously reported that NRG1/ErbB signaling is essential for proper metabotropic glutamate receptors 1 (mGluR1) functioning in midbrain DA neurons, thus the functional interaction between ErbB receptors and mGluR1 regulates neuronal excitation and *in vivo* striatal DA release. While it is widely recognized that mGluR1 play a pivotal role in long-term modifications of synaptic transmission in several brain areas, specific mGluR1-dependent forms of synaptic plasticity in substantia nigra pars compacta (SNpc) DA neurons have not been described yet. Here, first we aimed to detect and characterize mGluR1-dependent glutamatergic long-term depression (LTD) in SNpc DA neurons. Second, we tested the hypothesis that endogenous ErbB signaling, by affecting mGluR1, fine-tunes glutamatergic synaptic plasticity in DA cells. We found that either pharmacological or synaptic activation of mGluR1 causes an LTD of AMPAR-mediated transmission in SNpc DA neurons from mice and rat slices, which is reliant on endogenous NRG1/ErbB signaling. Indeed, LTD is counteracted by a broad spectrum ErbB inhibitor. Moreover, the intracellular injection of pan-ErbB- or ErbB2 inhibitors inside DA neurons reduces mGluR1-dependent LTD, suggesting an involvement of ErbB2/ErbB4-containing receptors. Interestingly, exogenous NRG1 fosters LTD expression during minimal mGluRI activation. These results enlarge our cognizance on mGluR1 relevance in the induction of a novel form of long-term synaptic plasticity in SNpc DA neurons and describe a new NRG1/ErbB-dependent mechanism shaping glutamatergic transmission in DA cells. This might have important implications either in DA-dependent behaviors and learning/memory processes or in DA-linked diseases.

## Introduction

Neuregulins (NRGs) are a family of epidermal growth factor (EGF)-related proteins acting as neurotrophic and differentiation agents. Besides being critically involved in the development of the central nervous system (CNS), increasing evidence demonstrates that NRGs represent important neuromodulators in the adult brain. NRGs are encoded by six genes (*NRG1–NRG6*), each producing numerous isoforms, all expressing an EGF-like domain required for the activation of the tyrosine kinase receptors of ErbB family. Four ErbB subtypes (ErbB1–4) have been identified, of which ErbB2, ErbB3 and ErbB4 mediate NRGs signaling, by constituting homo- and/or heterodimers upon NRGs binding, and activating different kinases pathways (Mei and Nave, 2014).

Several evidences support an important role for NRG1/ErbB signaling in the modulation of midbrain dopamine (DA) system. Actually, DA neurons express NRG1 and ErbB receptors, throughout development into adulthood (Steiner et al., 1999; Thuret et al., 2004; Abe et al., 2009; Namba et al., 2009; Zheng et al., 2009). Moreover, ErbB ligands have neurotrophic and neuroprotective effects on midbrain DA neurons. Thus, ErbB signaling activation fosters morphological/biochemical differentiation of immature DA cells (Casper et al., 1991; Ferrari et al., 1991;Casper and Blum, 1995;Farkas and Krieglstein, 2002; Zhang et al., 2004) and protects midbrain DA neurons from neurotoxin-induced degeneration either in neuronal cultures (Ostenfeld et al., 1999; Hanke et al., 2004; Zhang et al., 2004; Iwakura et al., 2005) or in mice models of Parkinson’s diseases (PD) (Carlsson et al., 2011; Depboylu et al., 2015). Accordingly, the genetic deletion of ErbB receptors or their ligands impairs DA neuronal development (Erickson et al., 1997;Blum, 1998) and reveals behavioral anomalies usually associated with altered DA transmission (Stefansson et al., 2002; Futamura et al., 2003; Golub et al., 2004; Skirzewski et al., 2017).

NRG1/ErbB signaling affects DA neurotransmission, by acutely adjusting extracellular DA levels (Yurek et al., 2004;Kwon et al., 2008; Ledonne et al., 2015; Skirzewski et al., 2017). Indeed, intracerebral NRG1 injection either in the substantia nigra pars compacta (SNpc) (Yurek et al., 2004) or in the projection areas of nigrostriatal and mesolimbic/mesocortical pathways, like striatum (Skirzewski et al., 2017), hippocampus (Kwon et al., 2008) and medial prefrontal cortex (mPFC) (Skirzewski et al., 2017) increases extracellular DA levels. Moreover, endogenous ErbB signaling inhibition in SNpc precludes striatal DA release induced by glutamate-activated nigral depolarization (Ledonne et al., 2015). Furthermore, NRG1 exposure in neonatal period, due to sub-chronic systemic administration, prompts a persistent hyper-DAergic state in the PFC in the adulthood (Kato et al., 2010), and peripheral NRG1 administration in adult mice augments striatal DA levels (Carlsson et al., 2011). However, it has been also reported that adolescent rodents injected with a pan-ErbB inhibitor have increased striatal DA levels in the adulthood (Golani et al., 2014).

While the involvement of NRG1/ErbB receptors in the modulation of DA transmission appears evident, the underlying cellular/molecular mechanisms are less clear. Systemic neonatal NRG1 exposure has been associated to DA neurons hyperactivation (increased spike bursting and spontaneous firing), possible due to a reduced GABAergic transmission (Namba et al., 2016). Moreover, a functional interplay between ErbB4 and the DA transporter (DAT) in DAergic terminals has been proposed as an indirect mechanism by which NRG1/ErbB4 signaling regulates extracellular DA levels in the projecting areas (Skirzewski et al., 2017).

Regarding a direct role for NRG1/ErbB signaling in the regulation of the midbrain DA system, we have previously reported that it finely tunes glutamatergic transmission in DA neurons, by specifically affecting metabotropic glutamate receptors 1 (mGluR1) functioning (Ledonne et al., 2015). Indeed, NRG1/ErbB signaling controls new-synthesis and membrane trafficking of functional mGluR1 in SNpc DA neurons, thus affecting DA levels in the striatum (Ledonne et al., 2015).

mGluR1, together with mGluR5, belongs to the group 1 mGluRs (mGluRI) subclass of metabotropic glutamate receptors that are canonically linked to the G_q/11_ heterotrimeric G proteins (Ferraguti et al., 2008). In SNpc DA neurons, mGluR1 and mGluR5 are both expressed, although higher levels have been reported for mGluR1 respect to mGluR5 (Testa et al., 1994; Hubert et al., 2001). Several functional evidences support a central role for mGluR1 in the modulation of midbrain DA system. Indeed, mGluR1 activation in SNpc DA neurons induces an inward current mediated by transient receptor potential channels (TRPC) (Guatteo et al., 1999; Tozzi et al., 2003; Ledonne et al., 2015), an outward current mediated by Ca^2+^-activated potassium channels (K_Ca_) (Fiorillo and Williams, 1998), as well as an increase in intracellular Ca^2+^ levels (Guatteo et al., 1999; Morikawa et al., 2003) and a facilitation of burst firing discharge (Prisco et al., 2002). Moreover, stimulation of nigral mGluR1 acutely increase DA release in the striatum of freely moving rats (Ledonne et al., 2015). Functional roles of mGluR5 in DA neurons are less characterized, although its activation contributes to DHPG-induced currents (Kramer and Williams, 2015; Ledonne et al., 2015).

It is largely accepted that mGluR1/5 are pivotal modulators of synaptic transmission, being involved in various forms of synaptic plasticity in several brain areas, including the hippocampus, dorsal and ventral striatum, mPFC and cerebellum (Collingridge et al., 2010; Lüscher and Huber, 2010). Regarding midbrain DA nuclei, a critical role for mGluR1 in the regulation of glutamatergic synaptic strength has been reported in DA neurons of the ventral tegmental area (VTA) (Bellone and Lüscher, 2006). The mGluR1-dependent long-term depression (LTD) in VTA is especially unmasked in synapses already potentiated by psychostimulants exposure (Mameli et al., 2007), thus being considered an endogenous mechanism to overcame excessive psychostimulants-induced plastic modifications of glutamatergic inputs to DA neurons (Lüscher and Huber, 2010).

Otherwise, the involvement of mGluR1 in long-term adjustment of excitatory synaptic strength in DA neurons of SNpc has been less characterized. Previous reports demonstrated that mGluRI activation depresses glutamatergic synaptic transmission in SNpc DAergic cells from rat midbrain slices (Bonci et al., 1997), but an analysis of potential long-term effects of mGluR1 activation on excitatory synaptic transmission is lacking.

To fill this gap in knowledge, we first aimed to characterize mGluRI-dependent long-term modifications of glutamatergic synaptic transmission in SNpc DA neurons, which has been induced by either chemical- or synaptic mGluRI activation. Then, in light of the evidence that NRG1/ErbB signaling is an endogenous regulator of mGluR1 function in SNpc DA neurons (Ledonne et al., 2015), and it modulates mGluRI-dependent LTD in the hippocampus (Ledonne et al., 2018), we also tested the hypothesis that endogenous ErbB signaling, by controlling mGluRI-dependent functions, fine-tunes glutamatergic AMPAR-mediated synaptic strength in midbrain DA neurons.

## Materials and Methods

### Experimental Animals

All procedures were carried out following the guidelines on the ethical use of animals from the Council Directive of the European Communities (2010/63/EU) and were approved by the Animal Care Committee of Santa Lucia Foundation (Authorization N° DM81-2014 PR). C57BL6/J mice and Wistar rats were bred in our facility and housed in a temperature- (23 ± 1°C) and humidity-controlled environment (45%–60% relative humidity), with a 12 h light/dark cycle (lights off at 7 p.m.). Animals were allowed to take food and water *ad libitum*.

### Midbrain Slice Preparation

Acute midbrain slices, used to perform electrophysiological experiments, were obtained following standard procedures, as described in Ledonne et al. (2012), with minor modifications.

Briefly, male C57BL6/J mice and Wistar rats (18–23 days old) were anesthetized with isoflurane and decapitated. The brain was rapidly removed from the skull and a tissue block containing the midbrain was isolated and immersed in cold artificial cerebrospinal fluid (aCSF) at 8–10°C. The aCSF contained (in mM): NaCl 126, KCl 2.5, MgCl_2_ 1.2, CaCl_2_ 2.4, NaH_2_PO_4_ 1.2, NaHCO_3_ 24, glucose 10, saturated with 95% O_2_–5% CO_2_ (pH 7.4). Horizontal slices (250 μm thick) of the ventral midbrain were cut using a vibratome (Leica VT1000S, Leica Microsystems, Wetzlar, Germany). Slices were maintained in aCSF at 33.0 ± 0.5°C for 30 min before being transferred in the recording chamber for the electrophysiological recordings.

### Electrophysiology

Whole-cell patch-clamp recordings of SNpc DA neurons were performed at 33.0 ± 0.5°C in a recording chamber placed on the stage of an upright microscope (Axioscope FS, Zeiss, Gottingen, Germany), equipped for infrared video microscopy (Hamamatsu, Tokyo, Japan). Slices were continuously perfused at 2.5–3.0 ml/min with aCSF. SNpc neurons, visually selected by their localization and morphology, were identified as DAergic based on the presence of regular spontaneous firing at 1.5–3 Hz (in cell-attached mode). Patch-clamp recordings were performed with glass borosilicate pipettes (6–8 MΩ) pulled with a PP-83 Narishige puller and filled with a solution containing (in mM): Cs-methanesulfonate 115, CsCl 10, CaCl_2_ 0.45, HEPES 10, EGTA 1, QX-314 5, MgATP 4, NaGTP 0.3 (pH 7.3 with CsOH). A bipolar parallel stimulating electrode (FHC Inc., Bowdoin, ME, USA) was placed rostral to the DA neurons recorded (100–200 μm). Excitatory postsynaptic currents (EPSCs) were evoked by delivering brief electrical pulses (100–200 μs duration, every 30 s) through a constant-current isolated stimulating unit (Digitimer, Welwey Garden City, UK). AMPAR-mediated EPSCs (AMPAR-EPSCs) were isolated by using the GABA_A_ receptor antagonist, picrotoxin (100 μM), the GABA_B_ receptor antagonist, CGP55845 (1 μM), the D2 receptor antagonist, sulpiride (1 μM), and the NMDAR blocker, MK-801 (10 μM). Amplitude and duration of stimulation pulses were set to obtain AMPAR-EPSCs of about 150–300 pA in baseline. A 2 mV hyperpolarizing step was continuously applied before each AMPAR-EPSCs to monitor changes in access resistance (R_a_). Recordings were discarded if R_a_ changed more than >20% during experiments or holding currents (to −70 mV) modified more than 100 pA during recordings.

The low frequency stimulation (LFS) protocol consisted in the delivery of pulses at the frequency of 1 Hz for 10 min (600 pulses), being the pulse amplitude and duration the same used for evoked AMPAR-EPSCs.

Otherwise stated, drugs were bath applied at known concentrations via a three-way tap system. A complete exchange of the solution in the recording chamber occurred in about 1 min. In a set of experiments, the pan-ErbB inhibitor PD158780 or the ErbB2 inhibitor, CP724714, as well as their vehicle (dimethyl sulfoxide, DMSO) were intracellular applied in SNpc DA neurons through the patch-clamp pipettes, during electrophysiological recordings. Thus, PD158780 and CP-724714 were dissolved at the final concentration of 1 μM in the pipette filling solution (1:10,000 in DMSO).

In another set of experiments, to investigate NRG1’s effect on mGluRI-dependent LTD, midbrain slices were kept in a holding chamber containing standard aCSF, saturated with 95% O_2_–5% CO_2_ at 33.0 ± 0.5°C, in presence of picrotoxin (100 μM), MK-801 (10 μM), CGP55845 (1 μM) and sulpiride (1 μM) (CTR), with the addition of NRG1 (5 nM, 30 min) or PD158780 + NRG1 (NRG1 5 nM plus PD158780 10 μM for 30 min preceded by PD158780 10 μM, 10 min), and then transferred in the recording chamber. Electrophysiological recordings on treated slices started within 30 min after the end of the treatment (slice removal from incubation chamber). Drug concentrations and durations of treatments were designed according to previous evidence (Ledonne et al., 2015, 2018).

AMPAR-EPSCs were analyzed by measuring peak amplitude and data were normalized to baseline. LTD magnitude was calculated by averaging AMPAR-EPSCs amplitude during last 5 min of recordings (i.e., 25–30 min after DHPG application).

### Statistical Analyses

Numerical data were expressed as mean ± SEM. Statistical comparisons of LTD magnitude were performed using Student’s unpaired *t*-test or One-way ANOVA, as appropriate.

### Drugs

Recombinant human NRG1β1 (EGF-like domain), sulpiride and isoflurane were purchased from Sigma-Aldrich (Milano, IT). PD158780 and CGP55845 were obtained from Tocris (Bristol, UK). (S)-DHPG, CP-724714, MK-801, Picrotoxin, CNQX, CPCCOEt and MPEP were from Abcam (Cambridge, UK).

## Results

### mGluR1-Dependent LTD of Glutamatergic Synaptic Transmission in SNpc DA Neurons

**Figure 1:**
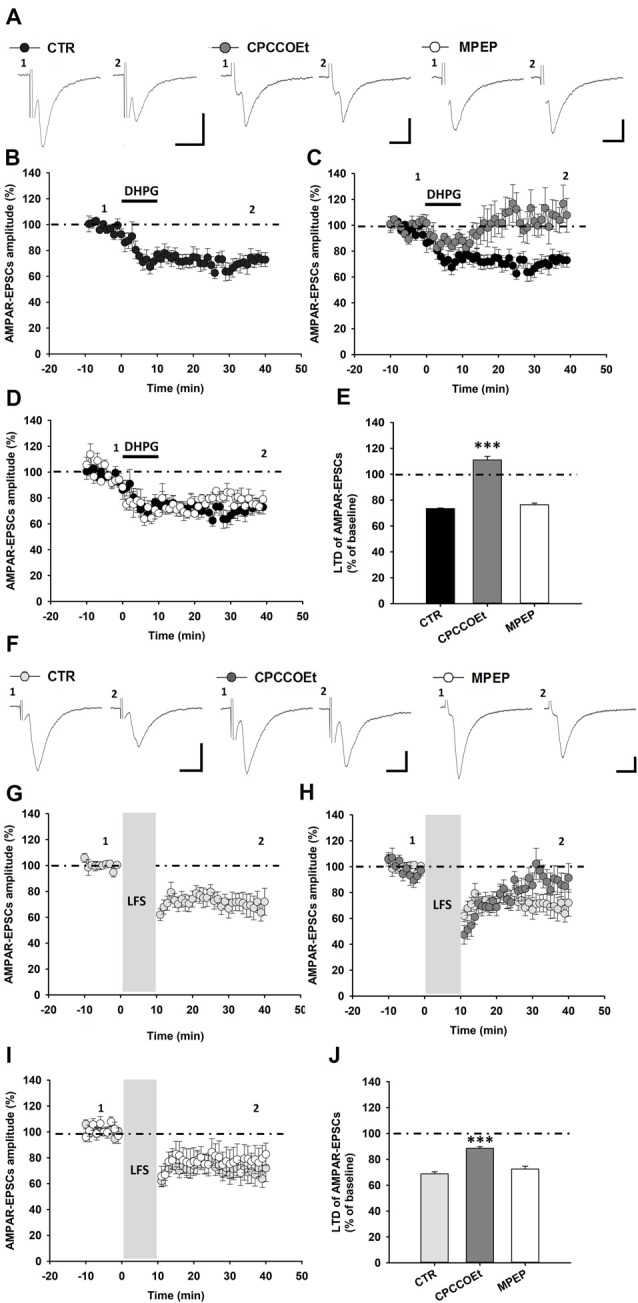
Metabotropic glutamate receptors 1 (mGluR1)-dependent long-term depression (LTD) in substantia nigra pars compacta (SNpc) dopamine (DA) neurons. **(A–E)** mGluRI activation with DHPG (100 μM, 10 min) induces an LTD of AMPAR-mediated excitatory postsynaptic currents (AMPAR-EPSCs) in SNpc DA neurons, which is dependent on the selective activation of mGluR1, but not mGluR5 receptors. **(A)** Representative traces of AMPAR-EPSCs showing the effect of DHPG (100 μM, 10 min) in control condition and in the presence of the mGluR1 antagonist, CPCCOEt (50 μM) or the mGluR5 antagonist, MPEP (10 μM) before (1) and after (2) DHPG application. The synaptic depression of DHPG-induced LTD **(B)** is counteracted by a pretreatment with CPCCOEt **(C)** but not MPEP **(D)**, as reported in the histogram **(E)** showing the magnitude of DHPG-induced LTD of AMPAR-EPSCs in the different pharmacological conditions. DHPG-induced LTD: CTR (*n =* 13 cells/9 mice), CPCCOEt (*n =* 7 cells/4 mice), MPEP (*n =* 5 cells/4 mice), ***p < 0.001 CPCCOEt vs. CTR, One-way ANOVA followed by Tukey’s test. **(F–J)** mGluR1 activation, by means of synaptically-induced glutamate release with a low frequency electrical stimulation (LFS), causes an LTD of AMPAR-EPSCs in SNpc DA neurons. **(F)** Representative traces showing AMPAR-EPSCs before (1) and after (2) the delivery of a protocol of LFS (1 Hz, 10 min) in control condition and in the presence of the mGluR1 antagonist, CPCCOEt or the mGluR5 antagonist, MPEP. **(G)** LFS-induced LTD is antagonized by a treatment with CPCCOEt (50 μM, 20 min before and during LFS) **(H)** but not MPEP (10 μM, 20 min before and during LFS) **(I)**, as showed in the histogram **(J)** reporting the magnitude of LFS-induced LTD in the different pharmacological conditions. LFS-induced LTD: CTR (*n* = 8 cells/5 mice), CPCCOEt (*n* = 6 cells/4 mice), MPEP (*n* = 7 cells/5 mice), ***p < 0.001 CPCCOEt vs. CTR, One-way ANOVA followed by Tukey’s test. **(A,F)** Scale bar: 100 pA, 5 ms.

To characterize the functional role of mGluRI in the regulation of excitatory synaptic plasticity in SNpc DA neurons, we performed patch-clamp recordings from these cells in midbrain slices of C57BL6 mice by analyzing the effect of an acute mGluRI stimulation, by means of the mGluRI agonist (S)-DHPG, on glutamatergic AMPARs-mediated synaptic transmission. We found that bath application of (S)-DHPG (100 μM, 10 min) induces an LTD of AMPAR-mediated excitatory postsynaptic currents (AMPAR-EPSCs) in SNpc DA neurons, being AMPAR-EPSCs mean amplitude reduced to 73.40 ± 0.48% of baseline (*n* = 13 cells/9 mice), at 25–30 min after DHPG exposure (Figures 1A,B,E).

To verify the selective involvement of mGluR1, rather than mGluR5, in DHPG-induced LTD, we analyzed LTD magnitude in midbrain slices treated with the selective mGluR1 antagonist, CPCCOEt or the mGluR5 antagonist, MPEP, respectively. Notably, DHPG-induced LTD was prevented by a pre-treatment with CPCCOEt, being not affected by MPEP (Figures 1A,C,D). Indeed, in slices treated with CPCCOEt (50 μM) for 20 min before and during DHPG application AMPAR-EPSCs mean amplitude was 111.00 ± 2.79% of baseline (*n* = 7 cells/4 mice, *p* < 0.001; Figure 1E) whereas in slices treated with MPEP (10 μM) for 20 min before and during DHPG AMPAR-EPSCs amplitudes were reduced to 79.77 ± 1.27% of baseline (*n* = 5 cells/4 mice, *p* > 0.05; Figure 1E).

The application of a prolonged low frequency electrical stimulation (LFS) determines a mGluRI-dependent LTD of glutamatergic synaptic transmission in different brain areas, as a consequence of extrasynaptic mGluR1/5 activation, by means of endogenous glutamate spillover (Bellone and Lüscher, 2006;Volk et al., 2006). To verify whether LFS could induce, by mGluR1, an LTD in SNpc DA neurons, we analyzed AMPAR-EPSCs amplitude while applying a classical LFS protocol (1 Hz, 600 pulses). We found that LFS delivery produced an LTD of AMPAR-mediated synaptic transmission in SNpc DA neurons (Figures 1F,G), reducing AMPAR-EPSCs mean amplitude to 68.81 ± 1.56% of baseline (*n* = 8 cells/5 mice; Figure 1J). LFS-induced LTD was reliant on selective mGluR1 activation, since it was counteracted by a pre-treatment with the mGluR1 antagonist CPCCOEt (Figures 1F,H), but not influenced by the mGluR5 antagonist MPEP (Figures 1F,I). Actually, following LFS delivery AMPAR-EPSCs mean amplitude was reduced to 88.49 ± 1.28% of baseline in SNpc DA neurons from slices treated with CPCCOEt (50 μM) for 20 min before and during LFS (*n* = 6 cells/4 mice, *p* < 0.001, unpaired *t*-test; Figure 1J) while its amplitude was 72.53 ± 2.11% of baseline in DA cells from slices treated with MPEP (10 μM) for 20 min before and during LFS protocol (*n* = 7 cells/5 mice; *p* > 0.05; Figure 1J).

Altogether, these results support a major role for mGluR1, rather than mGluR5, in the modulation of synaptic strength in SNpc DA neurons, being mGluR1 critically involved in either pharmacological or synaptic forms of LTD of AMPAR-mediated synaptic transmission.

### Endogenous ErbB Signaling-Dependent Regulation of mGluR1-Induced LTD in DA Neurons

**Figure 2:**
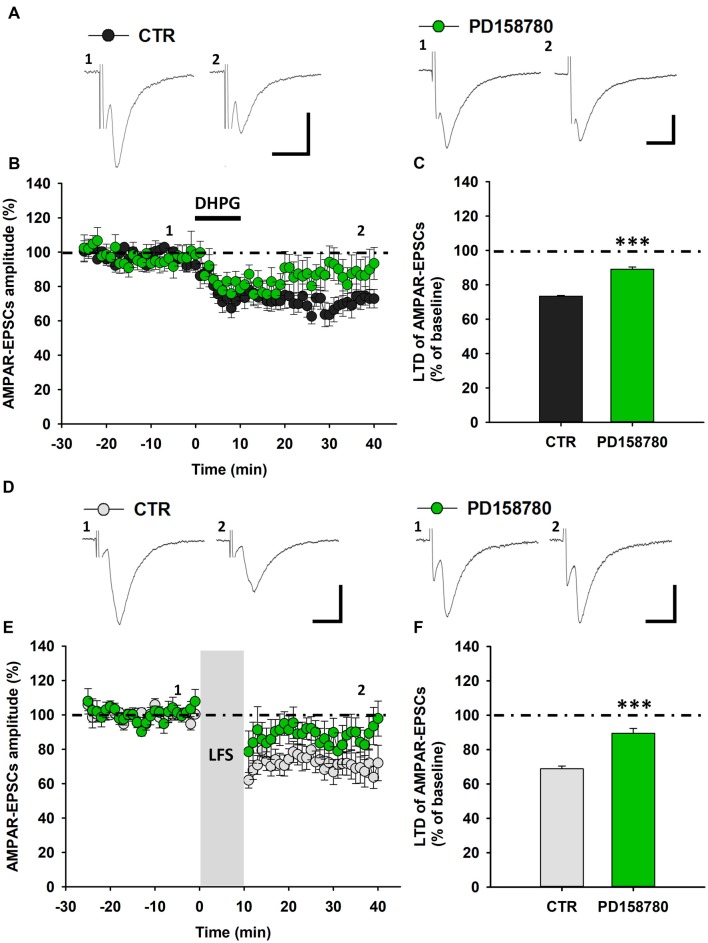
ErbB signaling inhibition counteracts mGluR1-dependent LTD in SNpc DA neurons from C57BL6 mice. **(A–C)** DHPG-induced LTD is antagonized by a treatment with the broad spectrum ErbB inhibitor PD158780. **(A)** Representative traces showing AMPAR-EPSCs before (1) and after (2) DHPG application in control conditions and in the presence of the pan-ErbB inhibitor, PD158780. **(B)** Time course of DHPG-induced LTD of AMPAR-EPSCs in control- and PD158780-treated slices. **(C)** Plot showing that the magnitude of DHPG-induced LTD is significantly reduced by a pretreatment with PD158780 (10 μM, 20 min before and during DHPG application). CTR (*n* = 13 cells/9 mice) and PD158780 (*n* = 7 cells/6 mice), ***p < 0.001. **(D–F)** LFS-induced LTD is counteracted by PD158780, as showed in the representative traces of AMPAR-EPSCs before (1) and after (2) the delivery of an LFS protocol (1 Hz, 10 min; **D**) and plots of time course **(E)** and magnitude **(F)** of LFS-induced LTD in control conditions and in slices treated with PD158780 (10 μM, 20 min before and during LFS delivery). CTR (*n* = 8 cells/5 mice) and PD158780 (*n* = 7 cells/5 mice), ***p < 0.001). **(A,D)** Scale bar: 100 pA, 5 ms.

To investigate whether NRG1/ErbB signaling affects mGluR1-dependent synaptic plasticity in SNpc DA neurons, we analyzed DHPG-induced LTD in control condition and after NRG1/ErbB signaling modulation. First, we aimed to determine the role of endogenous NRG1/ErbB tone, by evaluating the effect of a treatment with the broad spectrum ErbB inhibitor, PD158780, on DHPG-induced LTD. We found that a pre-treatment with PD158780 (10 μM), 20 min before and during DHPG application significantly blunted DHPG-induced LTD (Figures 2A–C). Indeed, in SNpc DA neurons from PD158780-treated slices, AMPAR-EPSCs mean amplitude, at 25–30 min after DHPG application, was 89.05 ± 1. 29% of baseline (*n* = 7 cells/6 mice, *p* < 0.001). PD158780 *per se* did not modified basal AMPAR-mediated transmission.

To further confirm the contribution of ErbB receptors in controlling mGluR1-dependent synaptic plasticity in SNpc DA neurons, we assessed the effect of PD158780 on LFS-induced LTD. Notably, PD158780 (10 μM), applied 25 min before and during LFS, similarly counteracted LTD of AMPAR-mediated synaptic transmission (Figures 2D–F), since AMPAR-EPSCs amplitude in SNpc DA neurons recorded from PD158780-treated slices was 89.44 ± 2.85% (*n* = 7 cells/5 mice, *p* < 0.001).

**Figure 3:**
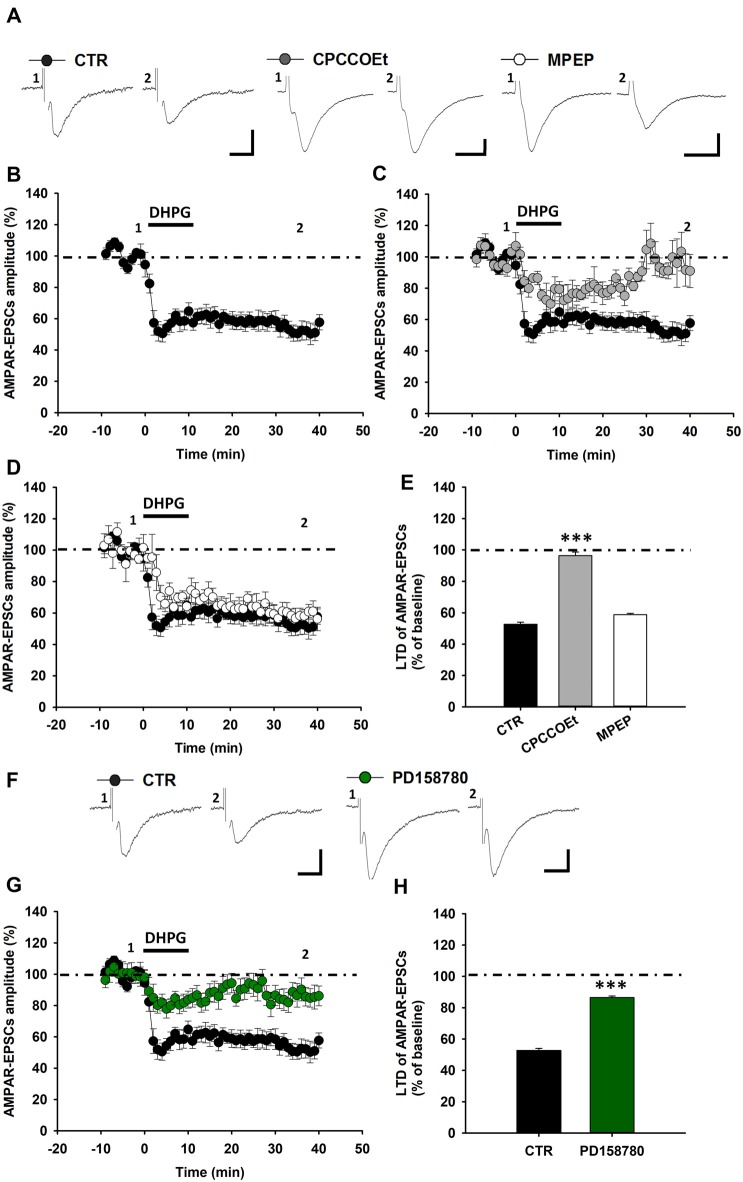
ErbB signaling inhibition affects mGluR1-dependent LTD in SNpc DA neurons from Wistar rats. **(A–E)** DHPG-induced LTD in SNpc DA neurons from Wistar rats. **(A)** Examples of AMPAR-EPSCs before (1) and after (2) the application of DHPG (100 μM, 10 min) in control condition and in slices treated with CPCCOEt (100 μM) or MPEP (10 μM). DHPG-induced LTD **(B)** is antagonized by a pretreatment with CPCCOEt **(C)**, while is not affected by MPEP **(D)**, as reported in the histogram **(E)** showing magnitudes of DHPG-induced LTD of AMPAR-EPSCs in the different pharmacological conditions. CTR (*n* = 8 cells/6 rats), CPCCOEt (*n* = 5 cells/5 rats), MPEP (*n* = 5 cells/5 rats). ***p < 0.001 CPCCOEt vs. CTR, One-way ANOVA followed by Tukey’s test. **(F–H)** DHPG-induced LTD is reliant on endogenous ErbB signaling activation in midbrain slices from Wistar rats.** (F)** Examples of AMPAR-EPSCs before (1) and after (2) the application of DHPG (100 μM, 10 min) in control condition and in slices treated with PD158780 (10 μM, 20 min before and during DHPG). **(G,H)** Plots of time course **(G)** and magnitude **(H)** of DHPG-induced LTD in different pharmacological conditions. CTR (*n* = 8 cells/6 rats) and PD158780 (*n* = 8 cells/6 rats), ***p < 0.001. **(A,F)** Scale bar: 100 pA, 5 ms.

Next, we aimed to verify whether this ErbB-dependent modulation of mGluR1-activated LTD is a species conserved mechanism of regulation of glutamatergic synaptic strength in SNpc DA neurons. Thus, we analyzed the contribution of ErbB receptors in mGluR1-dependent LTD in midbrain slices from Wistar rats. As in C57BL6/J mice, the pharmacological activation of mGluRI, by DHPG, in rat midbrain slices, caused a robust reduction of AMPAR-EPSCs in DA neurons, with AMPAR-EPSCs amplitude being diminished to 52.73 ± 1.30% of baseline (*n* = 8 cells/6 rats; Figures 3A,B,E). We found that also in Wistar rats DHPG-induced LTD was reliant on the selective activation of mGluR1, being antagonized by a pretreatment with the mGluR1 antagonist, CPCCOEt (Figures 3A,C,E), but not affected by the mGluR5 antagonist, MPEP (Figures 3A,D,E). Indeed, AMPAR-EPSCs mean amplitude in CPCCOEt-treated slices was 96.32 ± 2.35% of baseline (*n* = 5 cells/5 rats) whereas in MPEP-treated slices was 58.81 ± 0.89% of baseline (*n* = 5 cells/5 rats; *p* < 0.001 CTR vs. CPCCOEt).

Notably, the inhibition of endogenous ErbB signaling, with PD158780, antagonized DHPG-induced LTD also in SNpc DA neurons of rats. Indeed, in rat midbrain slices treated with PD158780 (10 μM, 20 min before and during DHPG application) the mean amplitude of AMPAR-EPSCs was 86.52 ± 0.97% (*n* = 8 cells/6 rats, *p* < 0.001; Figures 3F–H).

**Figure 4:**
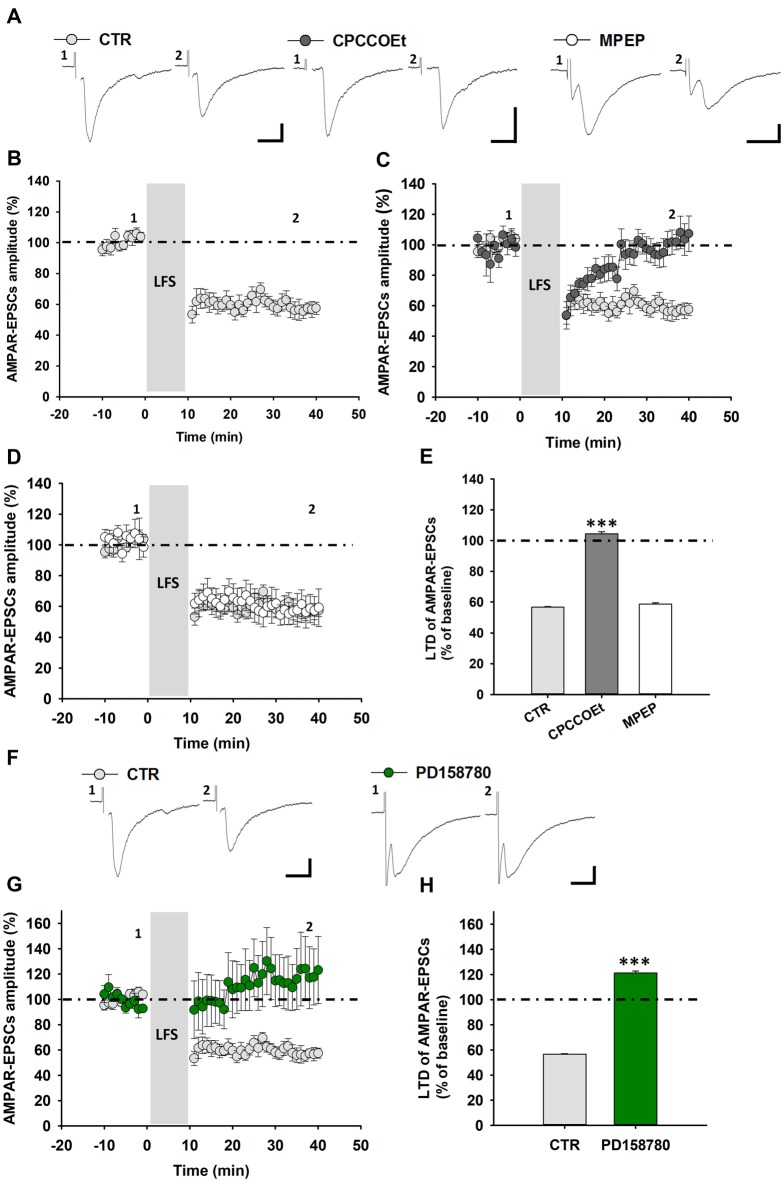
LFS-induced LTD in SNpc DA neurons from Wistar rats is affected by ErbB signaling inhibition. **(A)** Representative traces of AMPAR-EPSCs before (1) and after (2) the delivery of LFS (1 Hz, 10 min) in control conditions and in slices treated with the mGluR1 antagonist, CPCCOEt (100 μM, 20 min before and during LFS) or the mGluR5 antagonist MPEP (10 μM, 20 min before and during LFS). LFS delivery in midbrain slices from Wistar rats induces an LTD **(B)** which is dependent on the selective mGluR1 activation, since is counteracted by a pretreatment with CPCCOEt **(C)** but not MPEP **(D)**, as reported in the histogram **(E)** showing magnitudes of LFS-induced LTD in control conditions and in slices treated with CPCCOEt or MPEP. CTR (*n* = 7 cells/5 rats), CPCCOEt (*n* = 9 cells/7 rats), MPEP (*n* = 5 cells/4 rats). ***p < 0.001 CTR vs. CPCCOEt, One-way ANOVA followed by Tukey’s test.** (F–H)** LFS-induced LTD is prevented by the ErbB inhibitorPD158780, as showed in the representative traces of AMPAR-EPSCs **(F)** before (1) and after (2) the delivery of an LFS protocol (1 Hz, 10 min) and plots of time course **(G)** and magnitude **(H)** of LFS-induced LTD in control conditions and in slices treated with PD158780 (10 μM, 20 min before and during LFS delivery). CTR (*n* = 7 cells/5 rats) and PD158780 (*n = 5 cells/5 rats)*, ***p < 0.001. **(A,F)** Scale bar: 100 pA, 5 ms.

Then, we evaluated whether a synaptically-induced mGluR1-dependent LTD could be similarly elicited, with an LFS protocol, in rat SNpc DA neurons, being also reliant on ErbB receptors activation. Likewise, the delivery of an LFS protocol (1 Hz, 600 pulses) to rat midbrain slices induced an mGluR1-dependent LTD of AMPAR-mediated synaptic transmission in DA neurons (Figures 4A,B), causing a reduction of AMPAR-EPSCs mean amplitude to 56.66 ± 0.38% of baseline (*n* = 7 cells/5 rats; Figure 4E). Indeed, this LFS-induced LTD was completely blunted in the presence of CPCCOEt (100 μM), applied for 20 min before and during LFS (AMPAR-EPSCs mean amplitude 104.42 109.40 ± 1.36% of baseline, *n* = 9 cells/7 rats, *p* < 0.001, CTR vs. CPCCOEt; Figures 4A,C,E), but was not influenced by a pretreatment with MPEP (10 μM), applied 20 min before and during LFS (AMPAR-EPSCS mean amplitude 58.60 ± 0.86% of baseline, *n* = 5 cells/4 rats; Figures 4A,D,E), thus confirming herein, as in C57BL6/J mice, a prominent contribution of mGluR1 subtypes in this form of synaptic plasticity. Notably, a pretreatment with the pan-ErbB inhibitor PD158780 completely antagonized LFS-induced LTD and unmasked a potentiation of AMPAR-mediated synaptic transmission, following LFS delivery (Figures 4F–H). Indeed, AMPAR-EPSCs mean amplitude in PD158780-treated slices was 121.20 ± 1.57% of baseline (*n* = 5 cells/5 rats; *p* < 0.001; CTR vs. PD158780; Figure 4H).

Altogether, these results point to a conserved endogenous ErbB signaling-dependent mechanism of regulation of mGluR1-dependent LTD in SNpc DA neurons in the two different rodent species.

### Subunit Composition of ErbB Receptors Modulating mGluR1-Dependent LTD in DA Neurons

We previously reported that ErbB receptors containing ErbB2 and ErbB4 subunits are involved in NRG1-dependent regulation of mGluR1 functioning in rat SNpc DA neurons (Ledonne et al., 2015). To verify the contribution of these specific ErbB subunits in the modulation of mGluR1-dependent LTD in C57BL6/J mice, we applied ErbB inhibitors inside DA neurons through the patch-clamp pipettes, then recording DHPG-induced LTD. Indeed, we analyzed modifications in LTD magnitude in DA neurons injected with a broad spectrum ErbB inhibitor, PD158780, which inhibits either ErbB2 and ErbB4 subunits as well as the effects of the intracellular injection of a selective ErbB2 inhibitor, CP-724714.

**Figure 5:**
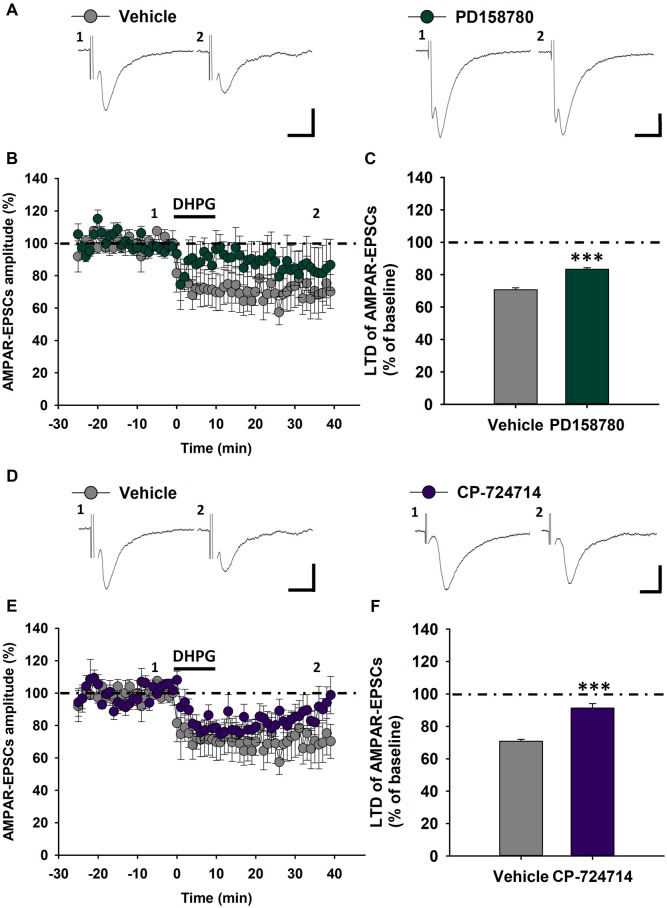
Effects of ErbB signaling inhibition inside single SNpc DA neurons on mGluR1-dependent LTD in C57BL6 mice. **(A–C)** The intracellular injection of the pan-ErbB inhibitor, PD158780 (1 μM), in SNpc DA neurons decreases mGluR1-dependent LTD respect to that induced by DHPG (100 μM, 10 min) in neurons injected with its vehicle (0.0001% DMSO). **(A)** Examples of AMPAR-EPSCs before (1) and after (2) DHPG application in Vehicle- and PD158780-injected cells. **(B,C)** Plots showing time course **(B)** and magnitude **(C)** of DHPG-induced LTD in Vehicle- and PD158780-injected neurons. Vehicle- (*n* = 5 cells/4 mice) and PD158780-injected neurons (*n* = 8 cells/6 mice), ***p < 0.001. **(D–F)** Effect of the ErbB inhibitor, CP724714 (1 μM) injected in single SNpc DA neurons on DHPG-induced LTD. **(D)** Representative AMPAR-EPSCs traces before (1) and after (2) application of DHPG in Vehicle- and CP724714-injected cells. **(E,F)** Plots of time course **(E)** and magnitude **(F)** of DHPG-induced LTD in Vehicle- and CP-724714-injected neurons. Vehicle- (*n* = 5 cells/4 mice) and CP-724714-injected neurons (*n* = 7 cells/6 mice), ***p < 0.001. **(A,D)** Scale bar: 100 pA, 5 ms.

We found that the intracellular application of PD158780 (1 μM) diminished mGluR1-dependent LTD. Indeed, the magnitude of LTD induced by DHPG (100 μM, 10 min) in PD158780-injected cells was significantly reduced respect to vehicle-injected cells (0.0001% DMSO; Figures 5A–C), being AMPAR-EPSCs mean amplitude 70.73 ± 1.18% of baseline in vehicle-injected cells (*n* = 5 cells/4 mice) and 83.31 ± 0.98% in PD158780-injected cells (*n* = 8 cells/6 mice, *p* < 0.001, Vehicle vs. PD158780). Similarly, LTD magnitude was significantly blunted in SNpc DA neurons injected with the ErbB2 inhibitor CP-724714, respect to vehicle-injected neurons (Figures 5D–F), being AMPAR-EPSCs mean amplitude in CP-724714-injected cells 91.26 ± 2.77% of baseline (*n* = 7 cells/6 mice, *p* < 0.001 vehicle vs. CP-724714).

These results support a key functional role of ErbB receptors, possible as ErbB2-ErbB4 dimers, specifically localized in SNpc DA neurons in the modulation of excitatory synaptic transmission in these neuronal population, by means of a specific regulation of mGluR1-dependent LTD.

### Exogenous NRG1 Increases mGluR1-Dependent Synaptic Depression in DA Neurons

**Figure 6:**
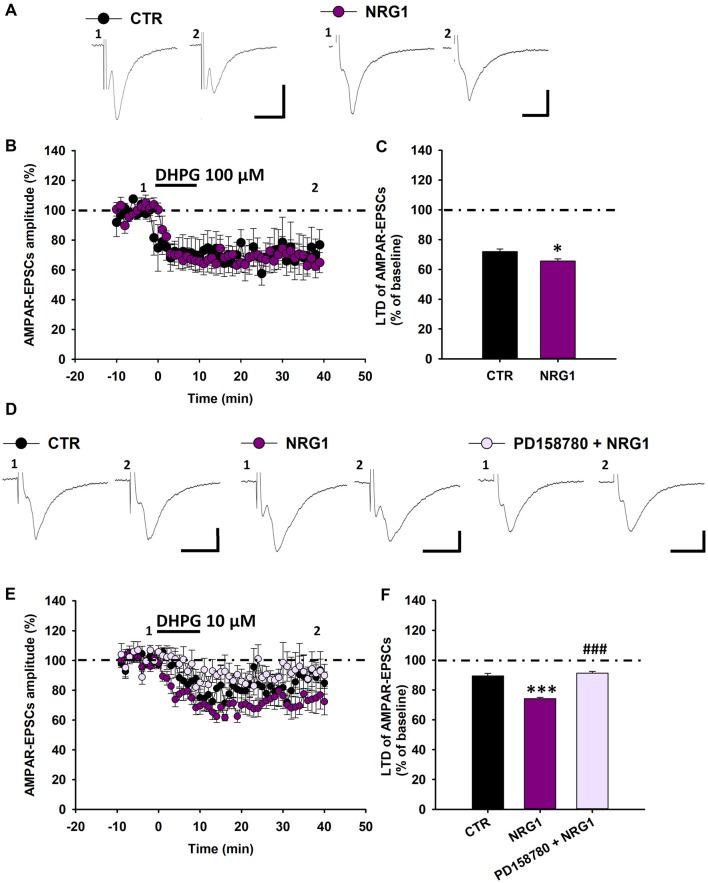
Exogenous NRG1 application fosters mGluR1-dependent LTD in DA neurons. **(A–C)** Effect of exogenous NRG1 on mGluR1-dependent LTD induced by DHPG (100 μM, 10 min). **(A)** Examples of AMPAR-EPSCs before (1) and after (2) the application of DHPG in control condition and in slices treated with NRG1 (5 nM, 30 min). **(B,C)** Plots showing time course **(B)** and magnitude **(C)** of DHPG-induced LTD. CTR (*n* = 5 cells/5 mice), NRG1 (*n* = 6 cells/5 mice), *p < 0.05. **(D–F)** Exogenous NRG1, through ErbB activation, gates mGluR1-dependent LTD by enhancing synaptic depression induced by low DHPG concentration (10 μM, 10 min). **(D)** Representative traces of AMPAR-EPSCs showing the effect of DHPG (10 μM, 10 min) in control condition, in slices treated with NRG1 (5 nM, 30 min) and in slices treated with PD158780 + NRG1 (PD158780 10 μM applied 10 min before and during NRG1 5 nM, 30 min). **(E,F)** Plots of time course **(E)** and magnitude **(F)** of DHPG-induced synaptic depression in different pharmacological conditions. CTR (*n* = 9 cells/5 mice), NRG1 (*n* = 9 cells/8 mice) and PD158780 + NRG1 (*n* = 6 cells/5 mice). ***p < 0.001 CTR vs. NRG1; ^###^*p* < 0.001 NRG1 vs. PD158780 + NRG1, One-way ANOVA followed by Tukey’s test.** (A,D)** Scale bar: 100 pA, 5 ms.

Then, we investigated whether exogenous NRG1 could affect mGluR1-dependent synaptic plasticity in SNpc DA neurons of C57BL6 mice. To this end we compared the magnitude of DHPG-induced LTD in midbrain slices treated with NRG1 (5 nM, 30 min) respect to control slices. We found that in NRG1-treated slices DHPG-induced LTD is marginally increased (Figures 6A–C), since AMPAR-EPSCs mean amplitude, following application of DHPG (100 μM, 10 min) was reduced to 65.57 ± 1.46% of baseline in NRG1-treated slices (*n* = 6 cells/5 mice) and to 71.98 ± 1.68% in control (CTR) slices (*n* = 5 cells/5 mice, *p* < 0.05, CTR vs. NRG1).

To possible unmask a more pronounced potentiating effect of NRG1 on mGluR1-dependent synaptic plasticity, we investigate the effect of NRG1 in condition of minimal mGluRI activation, which cause a minor synaptic depression in control conditions. We found that in NRG1-treated slices the synaptic depression induced by DHPG (10 μM, 10 min) is considerably potentiated (Figures 6D–F). Indeed DHPG (10 μM) reduced AMPAR-EPSCs mean amplitude to 89.44 ± 1.69% of baseline (*n* = 9 cells/7 mice) in CTR slices and to 74.22 ± 0.77% of baseline in NRG1-treated slices (*n* = 9 cells/8 mice; *p* < 0.001, CTR vs. NRG1). This NRG1-dependent potentiation of DHPG-induced LTD is mediated by the activation of ErbB receptors, since it was prevented by the pan-ErbB inhibitor PD158780. Indeed, in slices treated with PD158780 (10 μM) for 10 min before and during the incubation with NRG1 (5 nM, 30 min) AMPAR-EPSCs amplitudes, following DHPG application, were 91.21 ± 1.16% of baseline (*n* = 6 cells/5 mice, *p* < 0.001, NRG1 vs. PD158780 + NRG1; Figures 6D–F).

Overall, these results support an important role for NRG1-induced ErbB signaling activation in adjusting mGluR1-dependent LTD in midbrain DA neurons.

## Discussion

Here, we describe a novel form of mGluR1-dependent long-term synaptic plasticity in SNpc DA neurons in two different rodent species (mice and rats), and demonstrate that it is controlled by NRG1/ErbB signaling activation. In particular, we reported that either pharmacological or synaptic stimulation of mGluR1 causes an LTD of AMPAR-mediated synaptic transmission in SNpc DA neurons, which is reliant on endogenous ErbB activation tone within DA neurons. Thus, we disclose a new role for NRG1/ErbB signaling in the regulation of glutamatergic synaptic transmission in midbrain DA neurons.

Several evidences demonstrate that NRG1/ErbB signaling controls glutamatergic transmission in different brain areas, by means of various mechanisms which are area- and synapses-specific, being also related to the activation state of synapses. There is a general consensus that NRG1/ErbB signaling does not affect basal ionotropic glutamatergic transmission, since it does not modify AMPAR- or NMDAR-induced currents in hippocampal CA1 pyramidal neurons (Huang et al., 2000; Kwon et al., 2005; Bjarnadottir et al., 2007; Iyengar and Mott, 2008; Chen et al., 2010; Ledonne et al., 2018) as well as in cultured cerebellar granule neurons (Fenster et al., 2012) and in midbrain DA cells (Ledonne et al., 2015), but there is evidence that NRG1 reduces NMDAR-induced currents in cortical pyramidal neurons (Gu et al., 2005). Moreover, NRG1 influences glutamate uptake by increasing protein levels of excitatory amino acid carrier (EAAC1) in mPFC (Yu et al., 2015), thus directly affecting extracellular glutamate levels.

Consistent data indicate that NRG1/ErbB signaling affects long term potentiation (LTP) of glutamatergic transmission in the hippocampus (Huang et al., 2000; Roysommuti et al., 2003; Kwon et al., 2005; Agarwal et al., 2014) and amygdala (Jiang et al., 2013; Lu et al., 2014), thus, representing a critical pathway in downscaling synaptic strength in these brain areas (Mei and Nave, 2014). To this regard, we have recently demonstrated that NRG1/ErbB signaling also modulates glutamatergic LTD in the hippocampus, since it allows mGluRI-dependent LTD at CA3-CA1 synapses (Ledonne et al., 2018). In line with this, by showing that NRG1/ErbB signaling similarly controls mGluR1-mediated LTD in SNpc DA neurons, we highlight a role for NRG1/ErbB tone in the modulation of glutamatergic synaptic plasticity in midbrain DA cells.

### mGluR1-Dependent LTD in SNpc DA Neurons

mGluR1/5 are key players in the modulation of excitatory synaptic strength, and their activation induces a depression of glutamatergic synaptic transmission in several brain, including hippocampus, dorsal and ventral striatum, mPFC, cerebellum and VTA (Collingridge et al., 2010;Lüscher and Huber, 2010). Notably, our data demonstrating that either prolonged pharmacological and synaptic mGluR1 activation in SNpc DA neurons induces an LTD of AMPAR-mediated synaptic transmission extend the evidence of a central role for mGluR1 in the long-term regulation of glutamatergic synaptic strength in the brain. Accordingly, there is a previous observation of an acute depressant effect of mGluRI on the excitatory transmission (Bonci et al., 1997).

Differently from SNpc, pharmacological mGluR1 activation in VTA DA neurons triggers a transient synaptic depression in naïve synapses, whereas a sustained mGluR1-dependent LTD could be induced only in synapses potentiated by psychostimulants exposure. This LTD is reliant on mGluR1-induced modifications of AMPARs subunit compositions, which decrease ion channel conductances, thus weakening AMPAR-mediated transmission (Bellone and Lüscher, 2006; Mameli et al., 2007;Lüscher and Huber, 2010). Hence, our results suggest that area/neuronal population-specific differences exist in the threshold/sensitivity to mGluR1-dependent synaptic depression between SNpc and VTA DA neurons. These discrepancies could arise from different expression levels of mGluR1 in SNpc vs. VTA or to a more effective mGluR1 signaling in distinct DA neuronal populations, which allows long-lasting mGluR1-induced synaptic depression in SNpc DA cells also in naïve synapses. It should be also considered that differential experimental conditions used in previous electrophysiological recordings (mixed glutamatergic EPSCs vs. isolated AMPAR-EPSCs, or different filling electrode solutions, as well as variations in DHPG concentrations and treatment durations) might contribute to differences between mGluR1-dependent synaptic plasticity in VTA vs. SNpc DAergic neurons. Notwithstanding, a dissimilar NRG1 endogenous tone in SNpc vs. VTA might differently regulate mGluR1 levels in distinct DA neurons populations, thus producing differences in the expression of the mGluR1-induced LTD.

Regarding synaptically-induced LTD in midbrain DA neurons, previous evidence demonstrated that in SNpc/VTA DA cells the application of an LFS (1 Hz, 10 min) paired to neuronal depolarization during stimulation (V_hold_ −40 mV), triggers an LTD of glutamatergic transmission (mixed AMPAR- and NMDAR-activated currents; Jones et al., 2000; Thomas et al., 2000). The mechanisms underlying this form of LFS-induced LTD have been partially elucidated, indicating that it does not require activation of glutamatergic NMDA- or metabotropic receptors (Jones et al., 2000; Thomas et al., 2000). Rather, it is dependent on the activation of voltage-dependent Ca^2+^ channels (Thomas et al., 2000) and is negatively modulated by DA (Jones et al., 2000). In light of this previous evidence, and in the attempt to isolate a mGluR1-dependent synaptically-induced form of LTD of AMPAR-mediated transmission in SNpc DA neurons, we applied a classical LFS protocol (1 Hz, 10 min) in the absence of a neuronal depolarization (V_hold_ −70 mV), and in the presence of antagonists for GABA_A_ and GABA_B_ as well as DA D2, and NMDA receptors. Our results show that, in these conditions, a form of synaptically-induced LTD can be elicited that is dependent on mGluR1 activation, being counteracted by a pre-treatment with a selective mGluR1 antagonist, CPCCOEt, but not with the mGluR5 inhibitor MPEP.

Notably, despite our results demonstrate that mGluR1 activation represents an important shared mechanism triggering both types of synaptic plasticity, LFS delivery might also engage other mechanisms in addition to the selective activation of mGluR1, which might eventually account for the different time course of the antagonistic effect of CPCCOEt in the different types of LTD.

Notwithstanding, we have demonstrated that mGluR1 has a central role in the modulation of glutamatergic synaptic plasticity in SNpc DA neurons, being herein involved in either pharmacological or synaptic forms of glutamatergic LTD.

### NRG1/ErbB-Dependent Regulation of mGluR1-Induced Synaptic Plasticity

NRG1-activated ErbB signaling represents a critical pathway for proper mGluR1 functioning in midbrain DA neurons (Ledonne et al., 2015). Indeed, NRG1-dependent ErbB tone regulates expression levels and membrane trafficking of functional mGluR1 in SNpc DA neurons. Actually, the inhibition of endogenous ErbB signaling, by causing mGluR1 internalization, impairs mGluR1-dependent mechanisms on the nigrostriatal DA pathway, directly affecting DA neurons depolarization and *in vivo* striatal DA release (Ledonne et al., 2015).

In line with our previous evidence, here we have demonstrated that in SNpc DA neurons another important mGluR1 functional role (i.e., the induction of glutamatergic LTD) is compromised following ErbB inhibition, thus increasing the relevance of the interplay between NRG1/ErbB signaling and mGluR1 in these cells. Indeed, in midbrain slices treated with the pan-ErbB inhibitor, PD158780, either pharmacological or synaptic forms of mGluR1-dependent LTD (i.e., DHPG- or LFS-induced) were affected. Remarkably, our results suggest that the ErbB-dependent regulation of mGluR1-dependent LTD is a conserved mechanism controlling glutamatergic synaptic strength in midbrain DA neurons, being ErbB signaling essential for proper mGluR1-dependent LTD in different rodent species (either C57BL/6 mice or Wistar rats). Moreover, by pursuing an intracellular inhibition of ErbB receptors inside SNpc DA neurons, we confirmed that ErbB2, besides ErbB4 subunits, are involved in the modulation of mGluR1-dependent LTD, in line with a role for ErbB2-ErbB4 dimers in the regulation of mGluR1 trafficking in rat SNpc DA neurons (Ledonne et al., 2015). To this regard, we have recently reported a similar involvement of ErbB2 subunits in the modulation of mGluRI-dependent LTD in the hippocampus (Ledonne et al., 2018).

Besides determining the role of endogenous ErbB signaling on mGluR1-dependent LTD in SNpc DA neurons, we have analyzed whether an exogenous application of NRG1 could enhance/facilitate mGluR1-induced synaptic plasticity in DA neurons. Interestingly, we found that exogenous NRG1, through ErbB activation, fosters LTD expression in conditions of minimal mGluRI activation, thus indicating that this NRG1-dependent mechanism is involved in gating mGluR1-dependent synaptic plasticity in midbrain DA neurons.

Since we have recently demonstrated that endogenous NRG1/ErbB signaling similarly modulates mGluRI-dependent LTD in the hippocampus (Ledonne et al., 2018), the crosstalk between NRG1/ErbB signaling and mGluRI may be a shared mechanism of regulation of glutamatergic synaptic plasticity in the brain. Notably, NRG1 also impairs hippocampal mGluRI-dependent LTD of GABAergic transmission (Du et al., 2013). Thus, NRG1-dependent ErbB activation, by damaging glutamatergic LTP and favoring mGluRI-dependent glutamatergic LTD, represents a critical mechanism balancing LTP/LTD equilibrium, thus shaping strength of excitatory transmission in different brain areas.

Regarding the modulation of midbrain DA system, converging evidence suggests that NRG1/ErbB signaling acts as a positive modulator of DA transmission, since NRG1-dependent ErbB stimulation causes a hyperactivation of midbrain DA neurons by decreasing herein GABAergic inputs (Kato et al., 2010; Namba et al., 2016) and enhancing mGluR1-induced depolarizations (Ledonne et al., 2015). Thus, endogenous NRG1/ErbB signaling controls mGluR1-induced DA release in the striatum (Ledonne et al., 2015) and also shapes DA levels in projection areas by an ErbB4-dependent regulation of DAT (Skirzewski et al., 2017). Nonetheless, concerning the net contribution of the interaction between mGluR1 and ErbB receptors in the activation of midbrain DA system, it should be considered that while a brief stimulation of nigral mGluR1, which causes an inward current and fosters burst firing generation (Guatteo et al., 1999; Prisco et al., 2002), increases the phasic DA release in the striatum (Ledonne et al., 2015), a more prolonged mGluR1 activation, by inducing glutamatergic LTD, could potentially decreases the overall activity of SNpc DA cells, by rendering them less influenced by an AMPARs-mediated excitatory drive. Thus, the whole contribution of the ErbB-mGluR1 functional interplay on the regulation of midbrain DA system could be dependent on the duration of stimulation of mGluR1 (brief vs. prolonged) by endogenous glutamate. For these reasons, it could be possible that the NRG1/ErbB tone, regulating mGluR1 functions contributes to an accurate adjustment of tonic/phasic DA release in the striatum.

### Potential Physiopathological Implications and Conclusions

mGluR1-dependent LTD has a pivotal part in learning/memory processes and behaviors involving cerebellum, hippocampus, VTA and striatum (Collingridge et al., 2010;Lüscher and Huber, 2010). It is well established that the nigrostriatal DA pathway plays an important role in the establishment of goal-oriented behaviors, like feeding and locomotion as well as in different cognitive functions, including reward/aversion-based learning, mental flexibility and habit-formation (Da Cunha et al., 2002, 2006; Palmiter, 2008; Wise, 2009; Haber, 2014; Ilango et al., 2014; Ledonne and Mercuri, 2017). Thus, it could be hypothesized that synaptic plasticity-related mechanisms within SNpc DA cells (like mGluR1-dependent LTD) might contribute and/or underlie these brain processes.

Regarding a potential relationship between NRG1/ErbB signaling in midbrain DA neurons and learning processes potentially associated to mGluR1-dependent synaptic plasticity in SNpc, it has been reported that a selective ErbB4 deletion in DA neurons specifically impairs spatial/working memory (Skirzewski et al., 2017), which is similarly affected by either systemic administration of mGluR1 antagonists or by a neurotoxin-induced lesion of SNpc (Da Cunha et al., 2002, 2003; Miyoshi et al., 2002; Braga et al., 2005; Hsieh et al., 2010; Sy et al., 2010). Therefore, although a direct link between ErbB-dependent regulation of nigral mGluR1 and working memory is lacking, an interplay between mGluR1-dependent synaptic plasticity and ErbB signaling in learning mechanisms concerning the nigrostriatal pathway could be conceived. Moreover, the mGluR1-dependent LTD in nigral DA neurons might be also involved in motor learning, being mGluR1 in the nigrostriatal pathway also potentially implicated in this learning process (Conn et al., 2005; Lüscher and Huber, 2010; Hodgson et al., 2011).

Notably, increasing evidence supports the contribution of mGluR1-dependent mechanisms in the pathogenesis of neurological and psychiatric disorders, such as schizophrenia, PD, addiction and autism (Ferraguti et al., 2008; Lesage and Steckler, 2010;Lüscher and Huber, 2010; Herman et al., 2012), which are characterized by alterations in midbrain DA transmission and also supposed to be linked to NRG1/ErbB dysfunctions (Han et al., 2012; Iwakura and Nawa, 2013; Mei and Nave, 2014; Ikawa et al., 2017).

Actually, mGluR1 regulates the postnatal maturation of glutamatergic synapses on VTA DA neurons (Bellone et al., 2011). An impairment of mGluR1-dependent LTD in VTA DA cells has been observed in a mouse models of autism (Bariselli et al., 2016) and it has been associated to addiction-related behaviors (Lüscher and Huber, 2010). Interestingly, genetic evidence suggests an association between altered NRG1/ErbB signaling and drug of abuse dependance (Han et al., 2012) as well as autism (Yoo et al., 2015), thus indicating that a dysfunction in ErbB-dependent regulation of mGluR1-activated LTD might be a contributing neurobiological mechanism underlying these diseases. Moreover, an unbalance of mGluR1-dependent LTD, due to altered NRG1/ErbB signaling in SNpc DA neurons, could contribute to the dysfunctions in the nigrostriatal DA transmission occurring in PD and schizophrenia (Perez-Costas et al., 2010; Yoon et al., 2013; Ledonne and Mercuri, 2017; Weinstein et al., 2017). To this regard, is should be considered that NRG1 and ErbB receptors represent candidate susceptibility genes for schizophrenia (Mei and Nave, 2014), which has been also linked to an aberrant mGluR1 functioning (Gupta et al., 2005; Lesage and Steckler, 2010; Volk et al., 2010; Ayoub et al., 2012; Herman et al., 2012).

Further studies are necessary to translate the functional relevance of ErbB-dependent regulation of mGluR1-mediated LTD in SNpc DA cells in the control of DA-related behaviors and learning/memory processes, as well as to unveil the potential involvement of NRG1/mGluR1 interplay in pathogenesis of neurological and psychiatric disorders associated to dysfunction of the midbrain DA system.

## Author Contributions

AL conceived the project and designed the experiments, performed and analyzed electrophysiological recordings and wrote the manuscript. NM conceived the project and wrote the manuscript.

## Conflict of Interest Statement

The authors declare that the research was conducted in the absence of any commercial or financial relationships that could be construed as a potential conflict of interest.
